# Engaging with communities to generate demand and improve the uptake of eye care

**Published:** 2022-09-20

**Authors:** Yesunesan Devanesam, Suresh Kumar Rajaram

**Affiliations:** Faculty: Lions Aravind Institute of Community Ophthalmology, Madurai, India.; Senior Faculty: Lions Aravind Institute of Community Ophthalmology, Madurai, India.

**During the pandemic, when traditional eye camps could not take place, Lions Aravind Institute of Community Ophthalmology worked with 14 eye hospitals to plan and deliver innovative approaches to finding people in rural areas who need eye surgery.**

**Figure:**
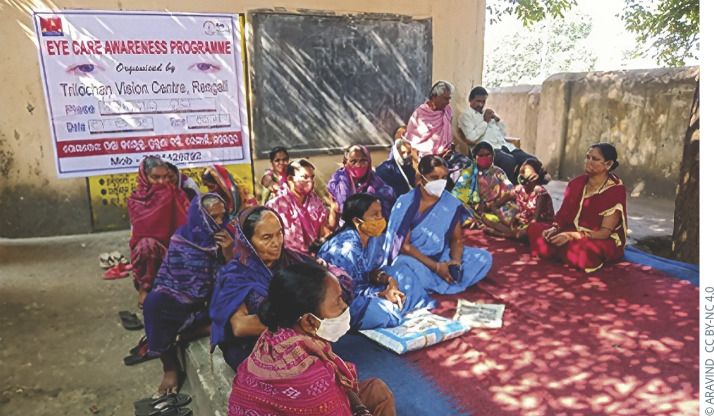
Health and community volunteers were trained to identify and refer people who need eye care. india

Vision loss is a major public health problem in low- and middle-income countries.^1^ Access to comprehensive eye care services is inadequate for people living in rural areas, compared to those living in urban and semi-urban areas. Access to eye care, and the costs associated with access, have long been reported as significant factors affecting the uptake of eye care services.^2^

Comprehensive eye screening camps have been widely used as a strategy to generate demand in rural areas and to overcome the challenges of access and affordability. However, the COVID-19 pandemic and the ensuing restrictions meant that eye screening camps could not be organised, which added considerably to the existing backlog of untreated eye conditions and low uptake of services. This made it necessary to identify and implement alternative strategies to find people who need eye surgery, particularly cataract surgery, and to encourage them to come to the hospital for surgery.

## Identifying strategies

Lions Aravind Institute of Community Ophthalmology (LAICO), the consultancy and training arm of Aravind Eye Care System, invited 14 partner eye hospitals from India, Bangladesh, Nigeria, and Kenya to a virtual brainstorming session to collectively propose and discuss different approaches to increase the demand for, and uptake of, eye care services.

Together, the group selected and refined a shortlist of six innovative approaches. Each hospital then chose one or more of the strategies to implement in their own setting. They considered:

- the resources (financial, human, and technological) available at the hospital
- the health infrastructure and human resources already available in the community
- the hospital's current level of engagement with various stakeholders in the community.

The initiative was carried out as an eight-month long collaborative mentoring programme organised by LAICO (from February to September 2021). A core implementation team of at least three members (hospital leader/ophthalmologist, hospital manager, and outreach coordinator) was formed at each hospital. A LAICO faculty member was then paired with each hospital to provide feedback once a week and to share an external perspective.

The hospital teams adapted their chosen approach (or approaches) to suit local conditions and developed the process flow, data collection templates, and monitoring mechanisms. Data from the field were captured using a simple Excel spreadsheet and integrated hospital management software (IHMS) was used to capture data in the hospital. WhatsApp was used as a communication tool between the community members and the hospital staff members.

Weekly reports were also shared with LAICO faculty members for individual feedback and discussion. LAICO organised monthly meetings involving all the hospitals to discuss the successes and challenges, which provided useful learning for everyone.

## Implementation

The six approaches are described in more detail below, with reference to the hospitals that adopted them.

### 1. Networking with health workers

Two hospitals in India – Srikiran Institute of Ophthalmology, Kakinada and Trilochan Netralaya, Sambalpur – adopted this approach. It involved training accredited social health activists and village health nurses – who were already working in rural communities to create awareness about health and support people's access to health-related services – to identify people with eye problems and refer them to the hospitals for further investigation and treatment. The hospitals put in place a referral tracking mechanism using a simple Excel spreadsheet saved on a computer at their hospital. They used it to record:

- the name of the person making the referral
- the name of the patient
- details about the referral
- the eventual diagnosis.

Both hospitals set up a system for receiving patients and providing treatment. Using the information recorded in the spreadsheet, hospital staff gave timely feedback to the referring health workers on their accuracy in identifying eye problems. This was done during meetings or by making individual phone calls. The referring health workers were paid for surgical referrals, according to current government guidelines.

Thanks to these referrals from community health workers, at the end of the eight-month period Srikiran Institute of Ophthalmology had carried out 926 cataract operations (21% of the total number carried out in the eight months), and Trilochan Netralaya had performed 601 cataract operations (10% of its total).

### 2. Referral by community organisations and volunteers

This method was adopted by three hospitals: Eye Foundation's Deseret Community Vision Institute in Ijebu-Imushin, Nigeria, Aravind Eye Hospital in Tirupur, India, and Sharda Netralay in Dhule, India.

It involved working with local community service organisations such as Lions Club, Rotary Club, a youth welfare club, and individual community members (such as village leaders or school teachers) and inviting them to refer patients with eye care needs to the hospital. Some of these organisations and community members had actively supported the hospitals’ outreach activities in the past.

Each hospital team held meetings with their local groups and individual volunteers to let them know that screening camps may not be held for some time due to the pandemic. They also explained that, compared to screening camps, their involvement in direct referral of patients would require less time, effort and money on the part of hospitals and the community.

Next, the hospitals handed out referral guidelines as well as referral cards in which volunteers could record patients' vital signs, such as their blood pressure and random blood sugar level (patients had to get these from their general medical practitioner). Blood pressure and blood sugar levels had to be known before patients could be referred, so that people wouldn't have to travel to hospital, only to find out they were not eligible for surgery due to systemic health issues.

The referral cards also had a space for the referrer to indicate whether or not they felt the patient required a free service. This was always accepted by the hospital, which indicates the high level of trust that existed between hospitals and the community organisations and volunteers. The hospital also kept the person who made the referral informed (via WhatsApp) about the patients' visit and their treatment at the hospital. The community volunteers reported feeling motivated by receiving such immediate feedback.

This approach accounted for 1,858 cataract operations over the eight months, which amounts to 18% of the total number of cataract operations the three hospitals carried out in that time.

Sharda Netralay had carried out 1,402 cataract operations; this was over 90% of the number of operations that resulted from their usual (and more expensive) methods of outreach before the COVID-19 pandemic. This was a strong encouragement for them to continue this approach in future.

### 3. Satisfied patients referring others

Patients tend to be happy if they have a good hospital experience and good visual outcomes, and satisfied patients can often be persuaded to refer other people in their community who also need surgery.

This approach was suggested by Kumudini Hospital (Bangladesh). It was adopted by 5 of the 14 hospitals: Kumudini Hospital (Bangladesh), Eye Foundation Deseret Community Vision Institute (Nigeria), and by Sharda Netralay, Sankara Eye Hospital, and Sitapur Eye Hospital (all three in India).

This approach could be implemented with almost no additional financial investment from the hospitals. It involved printing and handing out referral cards to satisfied patients and explaining how to use the cards to refer others with eye problems. Hospitals then sent a note of gratitude to the patients who referred people for treatment, which helped to motivate them to continue doing so.

As a result of this low-cost approach, a total of 1,014 cataract operations were performed by the five hospital, amounting to over 4% of the total number of cataract operations they performed in eight-month period. As a further step, Sharda Netralay was able to identify a few patients who became their regular outreach community sponsors; these patients now organise regular outreach camps in their respective communities.

### 4. Working with optometrists in the community

Midnapore Rotary Eye Hospital in India, one of the partner hospitals, reported having an arrangement with a local optometrist to refer patients who needed eye surgery. This idea was adapted to create a structured approach for involving optometrists in extending the reach of eye care services.

Netaji Eye Hospital, Purulia and Sharda Netralay also adopted this method. They identified several service-minded optometrists locally, had discussions with them, and set up a structured referral process that involved optometrists referring patients to the hospital for surgery, and the hospital referring patients back to the optometrists after surgery for spectacle prescriptions.

Optometrists were given guidance on:

- the type of patient to be referred
- the process of referring a patient to the hospital
- post-surgery follow-up care (including spectacle provision).

The hospitals gave feedback to the optometrists about each patient they referred and also held periodic review meetings with optometrists to monitor and improve their referral. This approach enabled the three hospitals to perform 1,710 cataract operations (11% of the total number of operations they carried out).

### 5. Village council (gram panchayat) outreach camps

Trilochan Netralaya suggested an innovative way of carrying out screening and referral camps in individual villages (a gram panchayat), which complied with the rules prohibiting large gatherings. The key differences were:

- Limited geographic coverage. Just one large village or two small villages were covered. In regular screening camps, all villages within 10–15 km of the camp location would have been covered.
- New ways of advertising. Volunteers from the village used local announcement channels to encourage patients to come for screening, rather than the more expensive, conventional hoardings (advertising boards), notices, or vehicle announcements.
- A smaller team. A three-member team – consisting of an optometrist or ophthalmic assistant, a field worker or outreach person, and a driver – visited the village by bus to screen the patients, instead of the usual eight or nine members in the case of a regular screening camp.

**Figure:**
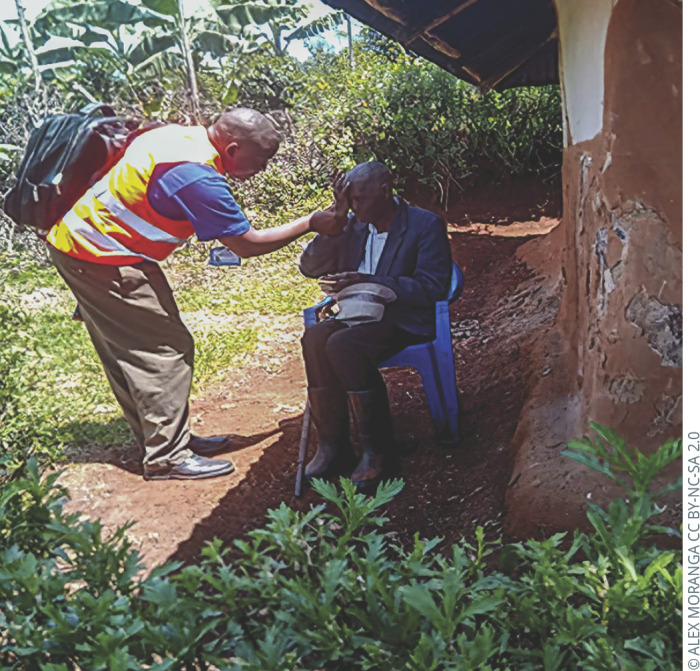
A cataract finder examines a patient. kenya

At times, the same three-member team was able to visit two sites in a single day, which would not be feasible in regular camps.

The hospital team transported those who needed cataract surgery to the hospital by bus, with social distancing measures and other COVID-19 protocols in place. The costs of the transport and community visits were paid by the respective hospitals. Patients with other eye problems were advised to visit the hospital on their own and seek treatment. Six hospitals adopted this approach, which resulted in 5,422 cataract operations (21% of the total number of operations they carried out).

### 6. Cataract finders

The objective of this approach was to recruit and train ‘cataract finders’ – local community members who are able to identify and refer cataract patients.

Kisii Eye Hospital, Kenya implemented this approach. The cataract finders were trained to assess visual acuity and recognise common eye diseases, including cataract. They were provided with motorcycles that allowed them to visit remote villages for screening. Each cataract finder had their own toolkit, containing a 6/18 visual acuity chart, a torch, measuring rope, and referral cards.

The cataract finders conducted door-to-door screening in their allotted villages, checked visual acuity, and identified patients with cataract. Screening data were documented in a prescribed format. Patients identified with cataract were referred to the hospital, and transport was provided for those who needed help.

**Figure:**
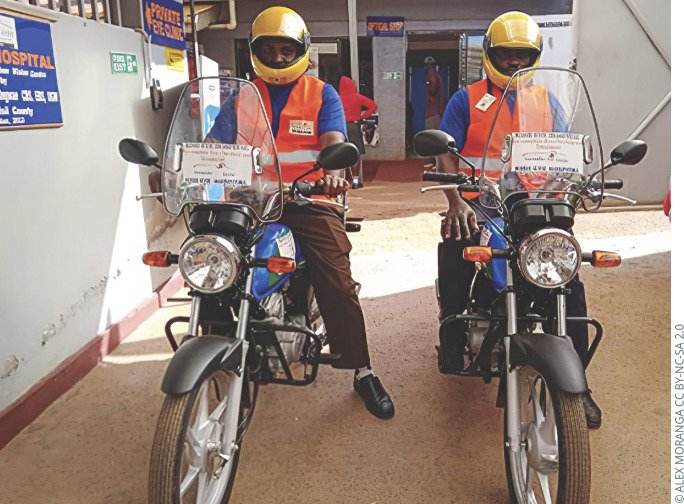
Cataract finders use motorcycles to reach remote villages. kenya

This approach resulted in 161 cataract operations being carried out at the hospital, accounting for just over 25% of the total for this period.

## Outcomes and next steps

Analysis of data from the 14 participating hospitals showed that the number of cataract operations made possible thanks to these six approaches contributed to almost 19% of the total number of cataract operations carried out across all the hospitals in the eight-month period (February to September 2021). This was possible because of the commitment of the hospitals to reach out to the community and the support of the people in the community.

None of the six approaches required much additional financial investment, and all 14 hospitals were able to implement at least one of them without needing additional staff members or resources. As a result, the hospitals were able to continue the strategies that worked well in their respective local conditions.

It is worth noting that all the strategies may not work well for every eye care provider. Local relevance and inherent or existing opportunities will determine the suitability of a strategy. Therefore, multiple approaches may have to be considered to create demand and enhance the uptake of eye care services.

In conclusion, responding to a health crisis (such as the COVID-19 pandemic) and ensuring continuity of care for those in need is a key responsibility of any health care provider. Innovative strategies and engagement with the community can help reach more people in need. Strategies that have proved to be effective need not be restricted to pandemic times but can be integrated into the mainstream to supplement the traditional outreach activities of the hospitals. Above all, ensuring the availability of services to every patient – from screening to postoperative follow-up – makes all the difference.
